# Photonic reservoir computing based on nonlinear wave dynamics at microscale

**DOI:** 10.1038/s41598-019-55247-y

**Published:** 2019-12-13

**Authors:** Satoshi Sunada, Atsushi Uchida

**Affiliations:** 1 Faculty of Mechanical Engineering, Institute of Science and Engineering, Kanazawa University, Kakuma-machi Kanazawa, Ishikawa, 920-1192 Japan; 2 Japan Science and Technology Agency (JST), PRESTO, 4-1-8 Honcho, Kawaguchi, Saitama 332-0012 Japan; 3 Department of Information and Computer Sciences, Saitama University, 255 Shimo-Okubo, Sakura-ku, Saitama City, Saitama 338-8570 Japan

**Keywords:** Information theory and computation, Optical physics, Statistical physics, thermodynamics and nonlinear dynamics, Information technology, Optics and photonics

## Abstract

High-dimensional nonlinear dynamical systems, including neural networks, can be utilized as computational resources for information processing. In this sense, nonlinear wave systems are good candidates for such computational resources. Here, we propose and numerically demonstrate information processing based on nonlinear wave dynamics in microcavity lasers, i.e., optical spatiotemporal systems at microscale. A remarkable feature is its ability of high-dimensional and nonlinear mapping of input information to the wave states, enabling efficient and fast information processing at microscale. We show that the computational capability for nonlinear/memory tasks is maximized at the edge of dynamical stability. Moreover, we show that computational capability can be enhanced by applying a time-division multiplexing technique to the wave dynamics. Thus, the computational potential of the wave dynamics can sufficiently be extracted even when the number of detectors to monitor the wave states is limited. In addition, we discuss the merging of optical information processing with optical sensing, revealing a novel method for model-free sensing by using a microcavity reservoir as a sensing element. These results pave a way for on-chip photonic computing with high-dimensional dynamics and a model-free sensing method.

## Introduction

Reservoir computing (RC)^1^, originally referred to as an echo state network^2^ or a liquid state machine^3^, is a computational paradigm that uses high-dimensional dynamical systems and has been regarded as a powerful tool for solving highly complex and abstract computational tasks. The computational paradigm has recently been implemented in a variety of physical systems and devices, such as optoelectronic systems^4^, photonic systems^5^, memristors^6^, spin systems^7^, and soft materials^8^. (See ref. ^9^ for a comprehensive review on physical RC). In particular, the photonic implementation of RC is expected to open the path to ultrafast and efficient processing beyond traditional Turing-von Neumann computer architecture^10–15^.

A key principle of the RC is high-dimensional mapping of the input information based on the high-dimensionality of the reservoir dynamical systems; the computational capacity is dependent on the number of linearly independent internal states of a dynamical system in response to an encoded input^16^. Moreover, nonlinearity and a short-term memory effect, inherent in dynamical systems, also play a crucial role in solving computational tasks requiring nonlinearity or memories. Thus, infinite dimensional nonlinear systems represent good candidates for use as reservoirs.

One such representative infinite dimensional system is a delay system, where reservoir networks can virtually be constructed in a time domain^17^. To date, numerous experimental studies of the photonic RC with delay systems have been performed because of the easy implementation in optoelectronic or photonic systems, such as lasers with delayed feedback^10,11,18,19^. The information processing, including prediction^10^ and speech recognition^11^, has been demonstrated. However, the drawback is the requirement of long delay lines to make many virtual nodes in the RC, which could lead to impractically large systems, inhibit stable operation, and may prevent practical deployment.

In this study, we propose the use of a microcavity laser, i.e., a microscale spatially extended optical system, as a reservoir. Originally, microcavities have mainly been utilized to realize a low threshold laser source and to modify quantum effects by implementing the strong optical confinement effect, which is caused by the difference in refractive indexes between inside and outside the cavity^20^. Furthermore, various shapes of microcavity lasers, inspired by wave/quantum chaos, have recently been utilized to control the emission properties^21,22^. An interesting feature of such microcavity lasers is the ability to exhibit a variety of spatiotemporal wave dynamics through the interplay of a gain medium and cavity shape^23,24^. Unlike previous work, we utilize such wave dynamics in microcavity lasers driven by an input signal for RC and numerically demonstrate that RC-based information processing can efficiently be achieved *at microscale* owing to the spatial degrees of freedom based on the high-dimensional dynamics with a long memory effect.

In addition, we discuss the application of microcavity-based processing by using the sensitivity of wave dynamics in a microcavity in an external perturbation. We propose the use of a microcavity as a sensing element as well as a reservoir, resulting in high-dimensional mapping. The merging of optical sensing and the reservoir suggests the possibility of novel sensing without complex post processing and theoretical sensing models. As a proof-of-concept demonstration, we show fast sensing of external reflective index by using the microcavity RC.

## Microcavity-based RC

**Figure 1:**
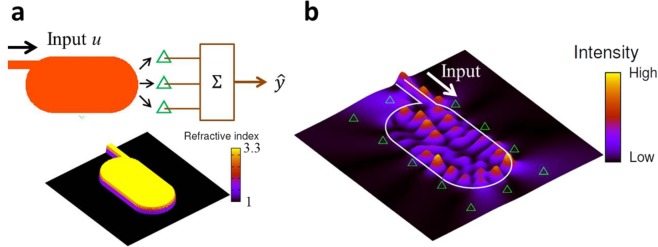
Microcavity laser for RC. (**a**) Schematic of RC using a microcavity laser. The cavity shape is designed as the Bunimovich’s stadium, known as a chaotic cavity, and the cavity is coupled to an input waveguide. The incident light encoded by the signal *u* is injected to the cavity. The emitted light intensities are detected by the probes, represented by the green triangles, and then used for the output $\hat{y}$. The lower figure in (**a**) shows the refractive index distribution of the stadium cavity coupled to an input waveguide. ${n}_{in}=3.3$ and ${n}_{out}=1.0$ denote the refractive indices inside and outside the cavity and waveguide, respectively. (**b**) An example of the intensity pattern responding to the input light, the frequency of which is tuned to a resonant frequency of the cavity ${\omega }_{0}$. The boundary of the cavity coupled to the waveguide is represented by the white curve. The green triangles represent the probe points to detect the intensity signals. In the simulation, the normalized pumping power ${W}_{\infty }/{W}_{th}\approx 1$ was set, where *W*_*th*_ is the threshold pumping power.

**Figure 2:**
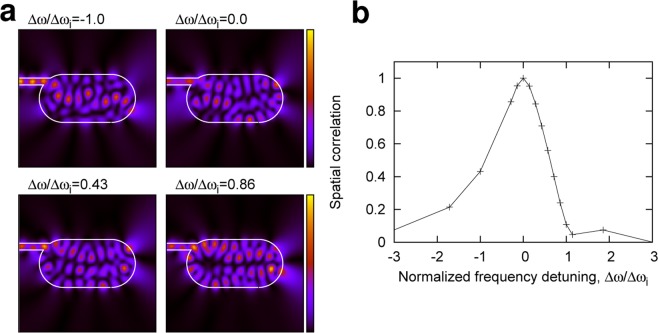
Dependence of intensity patterns on input frequency. (**a**) Examples of the intensity patterns in a stadium cavity. The intensity pattern changes according to the frequency detuning Δ$\omega$ of the input light from a resonant frequency ${\omega }_{0}$ of the cavity. Δ${\omega }_{i}$ denotes the average mode-interval of the cavity. (**b**) Spatial correlation between two intensity patterns with frequencies ${\omega }_{0}$ and $\omega ={\omega }_{0}+\Delta \omega$ is plotted as a function of the normalized frequency detuning $\Delta \omega /\Delta {\omega }_{i}$. The spatial correlation was calculated as $C(\omega )=\langle ({I}_{\omega }-{\bar{I}}_{\omega })\,({I}_{{\omega }_{0}}-{\bar{I}}_{{\omega }_{0}})\rangle /({\sigma }_{\omega }{\sigma }_{{\omega }_{0}})$, where 〈·〉 denotes the spatial average, and ${I}_{\omega }({\bf{r}})$ represents the intensity pattern for frequency $\omega$. ${\bar{I}}_{\omega ({\omega }_{0})}$ and ${\sigma }_{\omega ({\omega }_{0})}$ denotes the spatial average and standard deviation, respectively. In the simulation, ${W}_{\infty }=0$ was set.

Figure 1(a) shows a schematic of the proposed system, which consists of a microcavity coupled to an input waveguide and probes (detectors) to form the RC output $\hat{y}$. The microcavity includes a nonlinear gain medium, and the cavity shape is designed as the Bunimovich stadium^25^, in which ray orbits are proven to be fully chaotic and the corresponding wave patterns are complex (Fig. 1b). An optical signal encoded with a phase modulation is injected from the input waveguide and is able to reach all parts of the cavity owing to the chaotic multiple reflections at the cavity boundary while nonlinearly amplified by the gain medium. A feature of the stadium cavity is the frequency dependence of its wave pattern, resulting from multiple reflections and the resulting wave mixing (interference) in the cavity; the speckle-like wave pattern is sensitive to the input frequency (Fig. 2a). Actually, as demonstrated in Fig. 2b, the spatial correlation between two wave patterns excited by the input with frequencies ${\omega }_{0}$ and $\omega ={\omega }_{0}+\Delta \omega$ decreases as Δ$\omega$ increases. This means that the information can be encoded into the wave patterns with instant frequency by phase-modulating the input light. Moreover, the gain medium plays an important role in adding an additional nonlinearity and memory effect for the amplification. The emitted signals from the cavity are detected at the probe points with the sampling time interval ${\tau }_{s}$. In the simulation, *N* probes are assumed to be placed around the cavity.

For RC, we consider the linear readout $\hat{y}(t)={\sum }_{i=1}^{M}\,{w}_{i}{x}_{i}(t)$, where *x*_*i*_ is the detected intensity at probe *i*, ($i\in \{1,2,\ldots ,N\}$), at time $t=n{\tau }_{s}$ ($n\in \{1,2\ldots ,\infty \}$), and *w*_*i*_ is a readout weight. The goal of the processing is to approximate a functional relation between input signal $u(n)$ and target signal $y(n)$ using output $\hat{y}$. To this end, a finite set of training data ${\{u(n),y(n)\}}_{n=0}^{T}$ is utilized to determine the readout weights, such that the mean square error $1/T\,{\sum }_{n}\,|y(n)-\hat{y}(n){|}^{2}$ is minimized. In the training process, we simply use the least-squares method.

## Results and Discussions

To gain an insight into the computational capability of the microcavity-based RC, the numerical simulation was performed using the Maxwell-Bloch (MB) model, in which the gain medium is modeled as a simple two-level system^26^. Although the MB model is a simple model of microcavity lasers, the dynamical lasing phenomena can qualitatively be examined^27,28^. We assumed that the cavity is two-dimensionally extended on a plane, and that the electric field is polarized perpendicular to the plane. For generality, all variables were made dimensionless (see *Methods* for details), and we discuss the RC capability in reference to the dimensionless variables.

**Figure 3:**
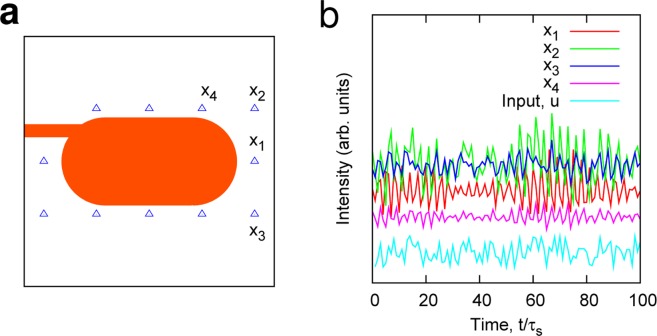
Demonstration of emission dynamics. (**a**) A schematic of the stadium-shaped microcavity laser coupled to an input waveguide. The blue triangles denote the probe positions. (**b**) Intensity signals *x*_*i*_ detected by the probes *i* = 1, 2, 3 and 4. The input light is phase-modulated with a signal *u*.

For the simulations, the refractive index *n*_*in*_ inside the cavity was set to be 3.3, and the length *L* of the major axis of the stadium cavity was ≈1.67*λ*, where *λ* is the wavelength of the input light in vacuum. (If *λ* = 0.85 *μ*m, *L* would be 1.42 *μ*m). ${\tau }_{s}$ was fixed to be close to the lifetime ${\tau }_{c}$ of the cavity without a gain medium. (See *Methods* for discussions concerning the actual experiments). The input information $u(n)$ was encoded in the input light phase as $\phi (t)={m}_{a}u(n)$, where ${m}_{a}\approx 0.1$ is the modulation amplitude. *u*(*n*) holds for the time interval ${\tau }_{s}$. The center frequency of the input light was locked to a resonant frequency ${\omega }_{0}$. Under these conditions, a variety of intensity signals were measured in response to the modulated signal, as demonstrated in Fig. 3.

### Nonlinear-memory capacity

**Figure 4:**
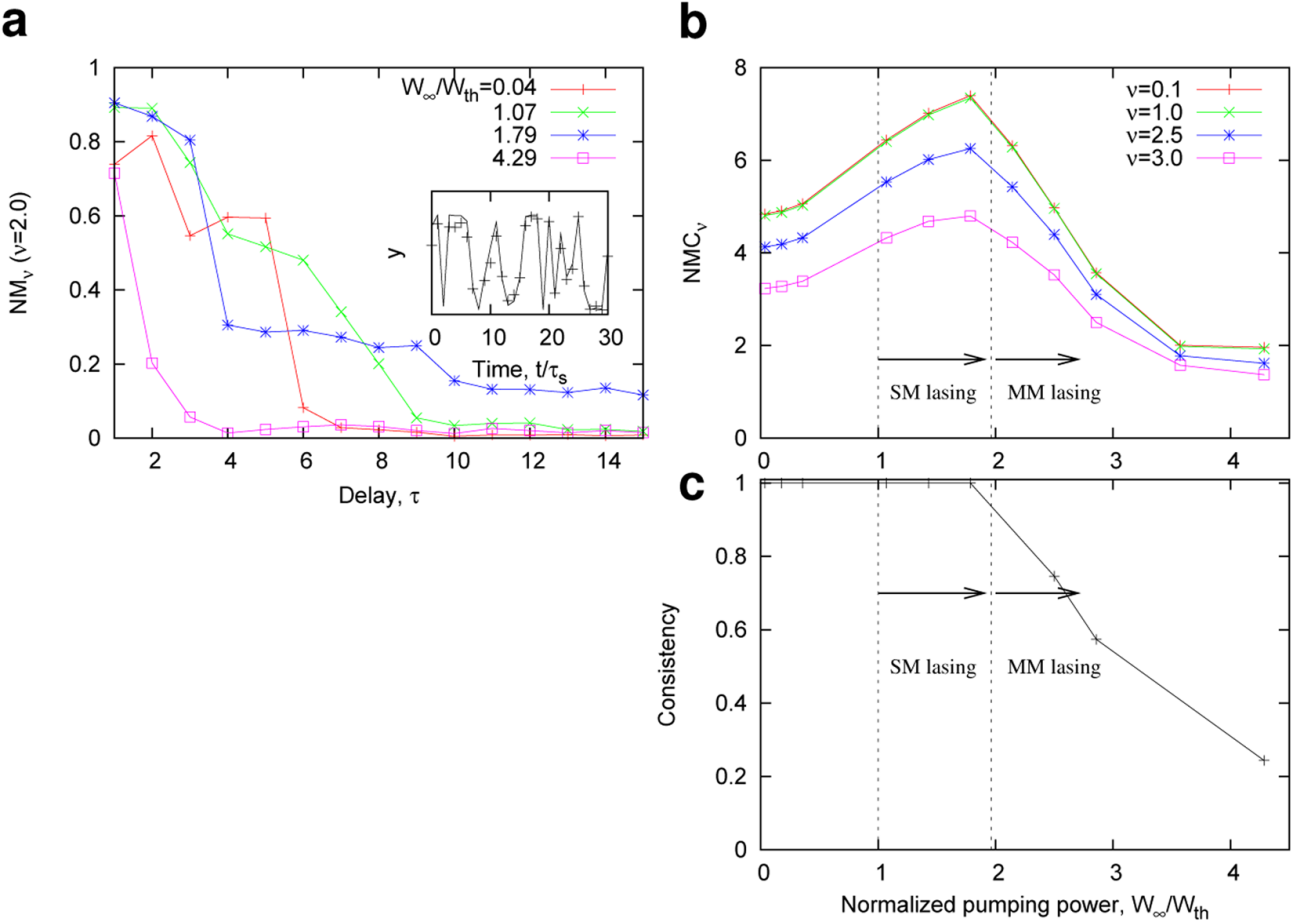
Nonlinear memory capacity of a stadium-shaped microcavity laser. (**a**) $N{M}_{\nu }(\tau )$ for $\nu =2.0$. ${W}_{\infty }/{W}_{th}$ denotes the pumping power normalized by the threshold pumping power. In the simulation, $N=11$ probes were used. The inset shows an example of the RC output $\hat{y}(n)$ for ${W}_{\infty }/{W}_{th}\approx 1.79$, denoted by the crosses, and the target signal *y*(*n*) for $\nu =2$ and $\tau =1$ (solid black curve) in time $t/{\tau }_{s}$. (**b**) *NMC*_*ν*_ as a function of the normalized pumping power ${W}_{\infty }/{W}_{th}$. When ${W}_{\infty }/{W}_{th} > 1$, a single mode (SM) lasing starts, and multimode (MM) lasing occurs when ${W}_{\infty }/{W}_{th} > 1.79$. For the large pumping power ${W}_{\infty }$, the loss of the input information is compensated, and a long memory effect can be achieved. In addition, nonlinear gain saturation plays an important role in introducing additional nonlinearity in the reservoir. However, consistency decreases when MM lasing occurs. Consequently, *NMC*_*ν*_ is maximized around the edge of the stability. (**c**) Consistency as a function of ${W}_{\infty }/{W}_{th}$. See *Methods* for the measurement method of the consistency.

For the evaluation of the computational capability, we used a simple function approximation task, $y(n)=\,\sin \,[\nu u(n-\tau )]/\nu$, where $\nu$ and $\tau$ are the task parameters that control the required nonlinearity and memory, respectively^29^. The input signal $u(n)$ is an identically distributed random sequence generated from a uniform distribution between $[\,-\,1,1]$. The goal of the task was to reproduce the nonlinearly converted signal $y(n)$ with a delay of $\tau$ (see the inset of Fig. 4a). To evaluate both the ability to adapt nonlinear tasks and memory capacity of the RC, we introduced the correlation between the target signal *y* and output $\hat{y}$,

1 $$N{M}_{\nu }(\tau )=\frac{{\langle y(n-\tau )\hat{y}(n)\rangle }^{2}}{{\sigma }_{y}^{2}{\sigma }_{\hat{y}}^{2}},$$

where 〈·〉 is the mean over time step *n*, *σ*_*z*_ denotes the standard deviation of $z=y$ or $\hat{y}$. Next, the nonlinear-memory capacity was defined as the sum of $N{M}_{\nu }(\tau )$, with $\tau$ to infinity:

2 $$NM{C}_{\nu }=\mathop{\sum }\limits_{\tau =0}^{\infty }\,N{M}_{\nu }(\tau ).$$

*NMC*_*ν*_ corresponds to the linear memory capacity *MC* in the limit of $\nu \to 0$^30^. With the nonlinear-memory capacity $NM{C}_{\nu \ne 0}$, one can evaluate both the nonlinearity and memory effects in microcavity lasers at the same time.

We examined the lasing dynamics in the stadium cavity and obtained *NMC*_*ν*_ from the intensity signals detected by each probe. In the simulation, $T=1000$ samples were used for the training, and the $N{M}_{\nu }(\tau )$ and *NMC*_*ν*_ were evaluated for the *T* samples. Figure 4 shows the numerical results of $N{M}_{\nu }(\tau )$ and *NMC*_*ν*_ with various values of the pumping power ${W}_{\infty }$ to activate the gain medium. (When ${W}_{\infty }=0$, the cavity is a passive cavity without gain. See *Methods* for ${W}_{\infty }$). As shown in Fig. 4a, $N{M}_{\nu }(\tau )$ decreases with increasing $\tau$, but the decrease becomes moderate by increasing ${W}_{\infty }$ in a range of $0\le {W}_{\infty }\le 1.79{W}_{th}$, where *W*_*th*_ denotes the threshold pumping power. The pumping compensates for the loss of the input information by the nonlinear amplification effect in the gain medium and the reservoir (cavity) can have a longer memory and becomes adaptive to nonlinear tasks. Accordingly, $N{M}_{\nu }(\tau )$ and the resulting *NMC*_*ν*_ increase with increasing ${W}_{\infty }$ (Fig. 4b). However, when ${W}_{\infty } > 1.79{W}_{th}$, multimode lasing occurs and *NMC*_*ν*_ decreases. Consequently, *NMC*_*ν*_ of the RC with the microcavity laser is maximized around the edge of the phase transition, ${W}_{\infty }/{W}_{th}\approx 1.79$.

The decrease in *NMC*_*ν*_ can be explained by the loss of the consistency^31^, or the so-called echo-state property^2^, which is an important condition for RC^29,32^. In the multimode lasing regime, the spontaneous multi-modal oscillations appear, leading to different results, even from the same input, depending on the initial state of the reservoir. Thus, the appearance of irreproducibility prevents consistent processing of the input information. We measured the consistency, defined as the mean correlation between the output signals starting from two different initial states. (see *Method* for further detail). As shown in Fig. 4c, the decrease in consistency is linked to the degrade of *NMC*_*ν*_. If consistency can be kept in the multimode lasing regime, better performance may be achieved through nonlinear mixing of different spatial modes.

As shown in the above-mentioned results, the effects of the gain medium, as well as the high-dimensionality of the wave states, play a crucial role in enhancing the computational capability of the RC frameworks. The compensation of the short memory, inherent in compact RC systems, and additional nonlinearity caused by the interaction with the gain medium are an advantage, compared to conventional *passive* photonic integrated RC^12,14,15^ where nonlinearity is introduced only as part of the measurement process.

### Effect of cavity shapes

**Figure 5:**
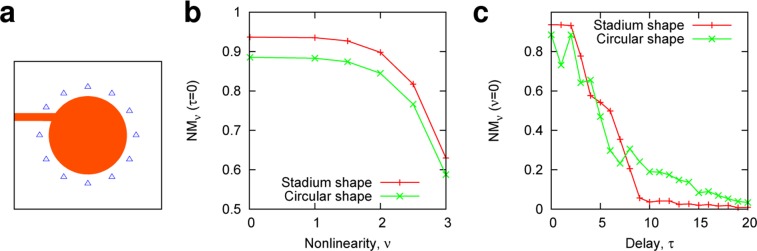
Performance comparison of stadium- and circular-shaped lasers. (**a**) A schematic of the circular-shaped laser coupled to an input waveguide. The blue triangles denote the probe positions. (**b**) Adaptivity to nonlinear tasks, where $N{M}_{\nu }(\tau =0)$ is shown as a function of the nonlinear parameter *ν*. (**c**) Linear memory capacity. $N{M}_{\nu =0}(\tau )$ is shown as a function of the delay parameter $\tau$. In (**b**,**c**), the performance of the stadium- and circular-shaped lasers are compared under the same input condition, same number of probes $N=11$, and same pumping condition in a single-mode lasing (consistency) regime. For nonlinear tasks, the *NM*_*ν*_ of the stadium-shaped laser is higher than that of the circular-shaped laser, as shown in (**b**). However, the circular-shaped laser outperforms the stadium-shaped laser for tasks requiring long memory $\tau \gg 1$, as shown in (**c**).

The cavity shapes play a crucial role in the quality of light confinement and the wave dynamics. In the stadium cavity, the chaotic multiple reflections lead to efficient wave mixing dynamics, enabling high-dimensional mapping of the input information into complex wave patterns, as demonstrated in the previous subsection. To gain a further insight into the effect of the wave-chaotic cavity on the RC performance, we also numerically examined the laser dynamics in a *non-chaotic cavity* where the internal ray orbits do not exhibit chaos. For the non-chaotic cavity, we chose a circular-shaped cavity (Fig. 5a) and compared the RC performance with that obtained in the stadium-shaped lasers with the same area and same pumping power condition in a consistency regime.

Figure 5b shows the performance comparison for nonlinear tasks, where $N{M}_{\nu }(\tau )$ is plotted as a function of the nonlinear parameter $\nu$ when the delay is $\tau =0$. Clearly, $N{M}_{\nu }(\tau =0)$ of the stadium-shaped laser can outperform that of the circular-shaped laser for all values of $\nu$, which may partly be attributed to a strong wave mixing effect in the stadium cavity. As shown in Fig. 5c, however, for tasks requiring memory with a delay parameter $\tau \gg 1$, the $N{M}_{\nu }(\tau )$ of the circular-shaped laser is relatively higher than that of the stadium-shaped laser because the circular cavity has a longer cavity-lifetime (lower loss rate)^22^. These results suggest a trade-off between the cavity shapes exhibiting a long-memory effect and nonlinearity. In terms of ray-wave correspondence^33^, the effect of the cavity shape becomes more dramatic for a larger value of a size parameter defined by ${n}_{in}L/\lambda$; it is expected to lead to a larger difference in the RC performance. The investigation concerning the aforementioned factors will be an important issue in the RC.

### The number of probes

In the microcavity-based RC, the number of the signals detected by the probes, *N*, corresponds to the number of reservoir nodes used to calculate the output $\hat{y}$, and it generally affects the capacity to approximate the target signal $y(t)$. To obtain a rich variety of responses from the reservoir nodes, the probes should at least be placed at a wavelength-scale distance from each other because shorter distance between the probes results in similar responses, i.e., ${x}_{j}\approx {x}_{i}$ $(j\ne i)$. This implies that the maximum number of the probes to be placed, *N*_*max*_, (effectively corresponding to the maximum number of nodes used to calculate $\hat{y}$) is limited by the cavity size and the wavelength. We roughly estimate *N*_*max*_ as the ratio of the perimeter of the stadium cavity, $P=(\pi /2+1)L$, to the characteristic wavelength $\lambda /{n}_{in}$ inside the cavity with *n*_*in*_, ${N}_{max}\sim (\pi /2+1){n}_{in}L/\lambda$, assuming that the spatial autocorrelation of the wave patterns is sufficiently small for a spatial scale larger than *λ*. For example, ${N}_{max}\approx 1,600$ nodes can potentially be used in a 0.013 mm^2^ footprint when *L* = 160 *μ*m, *λ* = 0.85 *μ*m, and ${n}_{in}=3.3$. We emphasize that the potential to implement such high-density and large-scale (virtual) nodes is unique to the wave dynamical RC and is not found in conventional photonic integrated RC^12^, comprised of multiple elements.

### Using spatiotemporal dynamics for RC

As mentioned above, the RC performance depends on the number of the probes *N* used for calculating the output $\hat{y}$. In an actual implementation, however, it may be practically difficult to place a large number of probes (or detectors) around a cavity. To overcome the problem, it should be noted that the dynamical information is included in delayed sequences, obtained from a few observables^34^. This suggests the possibility that, even when only a few observables are utilized, the dynamical information can be extracted from the dynamics of a few observables. In addition, the use of mask signals can create a rich variety of the responses from reservoir nodes^17^. We here use virtual nodes in a time domain for RC with the time-multiplexing method used in delay-based RC^17,30^. First, an input signal is multiplied by a mask signal with a period of *T*_*m*_. Then, each response *x*_*i*_ to the signal at probes *i* is sampled *M* times with a sampling interval ${\tau }_{s}(\,=\,{T}_{m}/M\approx {\tau }_{c})$. We describe *x*_*i*_ at time $t=n{T}_{m}+j{\tau }_{s}$ $(j\in [1,2,\ldots ,M])$ as the node labeled by *i* and *j*, i.e., ${x}_{ij}(n)={x}_{i}(n{T}_{m}+j{\tau }_{s})$. Moreover, we use the past node response ${x}_{ij}(n-k)$, ($k\in \{1,2,\ldots ,K\}$). Finally, the output signal $\hat{y}(n)$ at time step *n* is calculated as

3 $$\hat{y}(n)=\mathop{\sum }\limits_{i=1}^{N}\,\mathop{\sum }\limits_{j=1}^{M}\,\mathop{\sum }\limits_{k=0}^{K}\,{w}_{ijk}{x}_{ij}(n-k),$$

where *w*_*ijk*_ is an optimal weight obtained by using the least-squares method.

**Figure 6:**
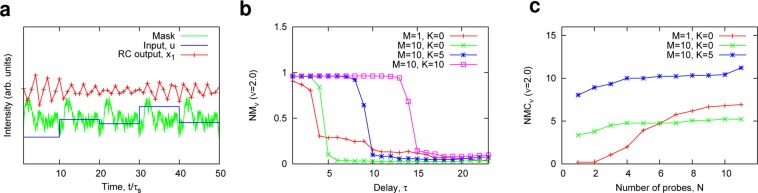
Information processing based on the time-multiplexing method. (**a**) An example of time-multiplexing encoding for $M=10$, where a random colored signal with period *T*_*m*_ is used as the mask signal. A variety of reservoir responses (for example *x*_1_) are obtained by the mask signal and input *u*. (**b**) $N{M}_{\nu }(\tau )$ as a function of the delay parameter $\tau$. $N{M}_{\nu }(\tau )$ improves by increasing both *M* and *K*. (**c**) *NMC*_*ν*_ vs. the number of probes *N*, corresponding to the number of the reservoir nodes. *NMC*_*ν*_ can increase as *N*, *M*, and *K* increase. Consequently, the large *NMC*_*ν*_ is achieved by the time-multiplexing method even when the number of probes is limited. In (**a**–**c**), ${W}_{\infty }/{W}_{th}=1.43$. In (**a**,**b**), $N=11$.

An example of the time-multiplexing method for $M=10$ is shown in Fig. 6a, where the input information *u* holds for the period *T*_*m*_, and the colored random signals with the period *T*_*m*_ are used as the mask signal because the use of colored noise, or chaotic oscillation, as the mask signals will lead to a good RC performance^35^. Figure 6b,c show the $N{M}_{\nu }(\tau )$ and *NMC*_*ν*_, respectively, for various values of *M* and *K*. When comparing the green curve ($M=10$ and $K=0$) to the red curve ($M=1$ and $K=0$) in Fig. 6b, one can see that $N{M}_{\nu }(\tau )$ increases for nonlinear tasks as *M* increases, when $\tau  < 5$. However, the large value of *M* results in decrease in memory, and $N{M}_{\nu }(\tau )$ for $M=10$ rapidly decreases for the tasks requiring the past information of $\tau  > 5$ (the green curve in Fig. 6b). Memory loss is compensated by increasing the number of past nodes *K*; thus, $N{M}_{\nu }(\tau )$ can be enhanced when *M* and *K* both increase, as shown by the blue curve ($M=10$ and $K=5$) and pink curve ($M=10$ and $K=10$) in Fig. 6b. We find that with the time-multiplexing method of $M=10$ and $K=5$, $NM{C}_{\nu }$ for only a single probe $N=1$ can be larger than *NMC*_*ν*_ without implementing the time-multiplexing method (Fig. 6c). The time-multiplexing method is effective in achieving high RC performance even when the number of the probes is limited. See Supplementary Information for further investigations on the RC performance obtained by the time-multiplexing method.

### Sensing applications

Physical RC frameworks generally suggest that physical systems responding to input signals *themselves* can be utilized as information processing systems. This implies that when physical systems are perturbed by an external stimulus (for example environmental changes), the system itself can be utilized to detect the external stimulus with an appropriate training process, in addition to monitoring internal states or observing unmeasured variables^36^. Here, we consider microcavities for the detection of an environmental physical quantity in the RC scheme and demonstrate the identification of a refractive index *n*_*out*_ outside the cavity, i.e., refractometric sensing.

**Figure 7:**
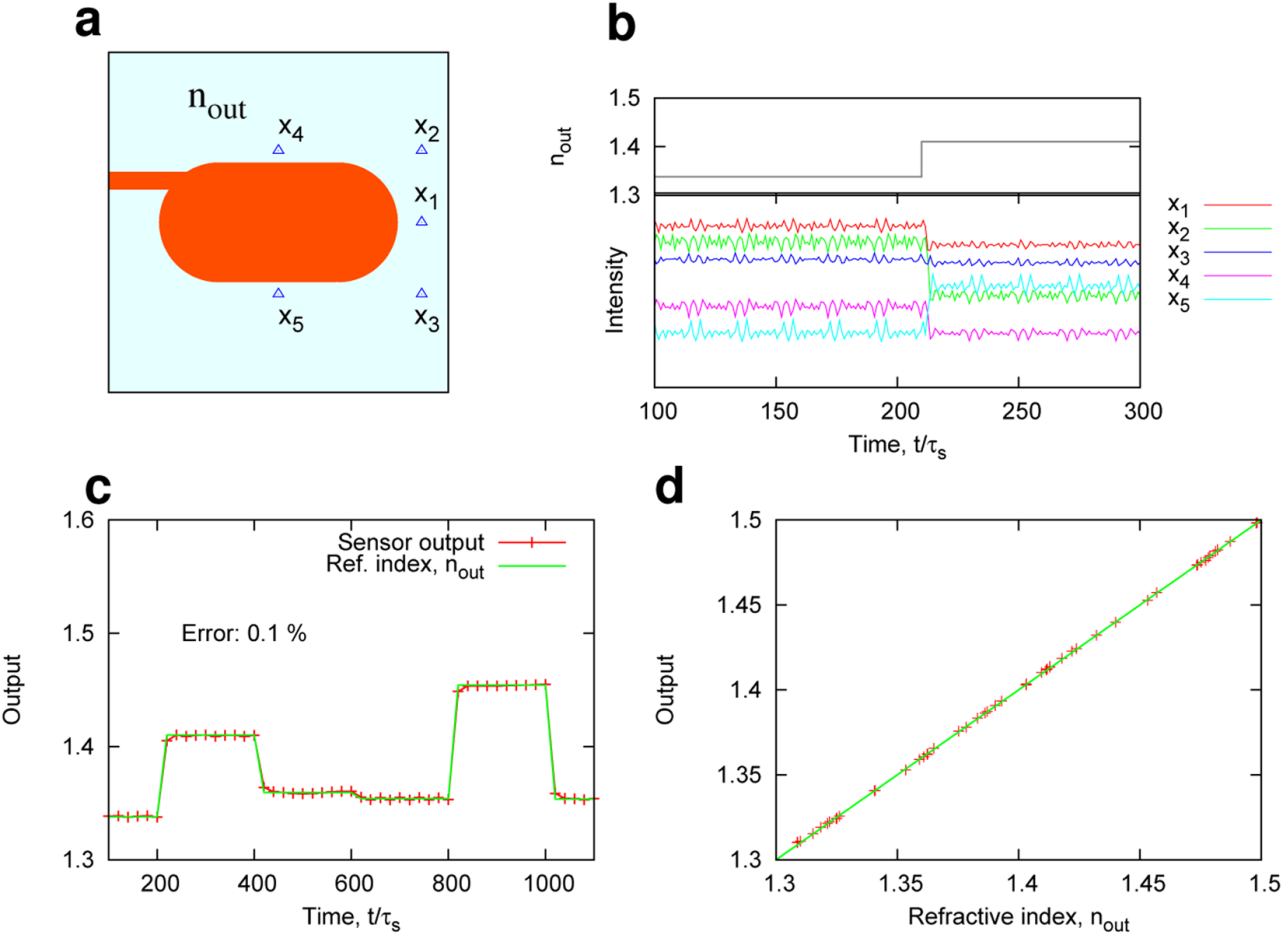
Sensing of environmental refractive index. (**a**) Stadium cavity used for the sensing of refractive index *n*_*out*_. The phase-modulated light is injected into the stadium cavity, and five signals detected by probes denoted by the blue triangles are used for the output $\hat{y}$. For passive cavity sensing, ${W}_{\infty }/{W}_{th}=0$ was set. (**b**) An example of the probe signal *x*_*i*_. *x*_*i*_ changes when *n*_*out*_ changes at time $t/{\tau }_{s}=215$. (**c**) The output signal $\hat{y}$ after training. $\hat{y}$ can identify *n*_*out*_, with 0.1% error, even when it rapidly changes. (**d**) The trained output $\hat{y}$ vs. refractive index *n*_*out*_.

As a simple demonstration, we consider the case when a stadium microcavity is surrounded by a medium with a refractive index *n*_*out*_, as shown in Fig. 7a. A randomly phase-modulated light is injected into the stadium cavity and emissions from the cavity are detected with the five probes ($N=5$). When the external refractive index *n*_*out*_ changes, the phases of the reflection/transmission and the coupling to the probes changes. Consequently, the detected intensity *x*_*i*_ at the probe *i* also changes (Fig. 7b). We use ${x}_{ij}(n)={x}_{i}(n{T}_{m}+j{\tau }_{s})$ ($j=1,\ldots ,M$) responding to the phase-modulated light to form $\hat{y}={\sum }_{i,j}\,{w}_{ij}{x}_{ij}$. Our purpose is to identify the surrounding refractive index *n*_*out*_ from the output $\hat{y}$ after the training of *w*_*ij*_ to minimize $\parallel {n}_{out}-\hat{y}\parallel$. In the training process, we used 100 datasets of $\{{x}_{1},\ldots ,{x}_{N},{n}_{out}\}$, where *n*_*out*_ was randomly chosen from a region of $1.3\le {n}_{out}\le 1.5$.

Figure 7c shows the trained output $\hat{y}$, where it is assumed that *n*_*out*_ randomly changes in time. Clearly, $\hat{y}$ follows the changes in the index *n*_*out*_ with the error of 0.1%, even when *n*_*out*_ rapidly changes in a timescale of ${\tau }_{s}\approx {\tau }_{c}$. Consequently, *n*_*out*_ can be identified with low errors, as shown in Fig. 7d. We remark that the memory of the reservoir (cavity) does not play an essential role in this sensing task. In this sense, the proposed learning-based sensing scheme is related to the extreme learning machine (ELM)^30,37^ as well as RC.

We emphasize that the proposed method does not need any precise sensing model, high-quality microcavity, or complex post processing, unlike previous work concerning microcavity sensors^38,39^, where the shift in the resonant frequency in a microcavity due to the change of the refractive index has been measured in accordance with the transmission or reflection spectra.

The results presented in this subsection suggest that in the merging of the optical sensing and learning-based processing, a model-free detection of the external refractive index *n*_*out*_ is achieved at a rate of $\mathrm{1/}{\tau }_{c}$.

## Summary

In this study, we have proposed and demonstrated RC based on nonlinear wave dynamics in a microcavity laser. An advantage of using a microcavity laser as a reservoir is that it can generate high-dimensional, nonlinear dynamics at microscale, enabling high-dimensional mapping of the input information to wave patterns after nonlinear amplification in the gain medium. We emphasize that the high-dimensional mapping to wave patterns is a spontaneous process with low energy loss, achieved by natural multiple reflections, at a short timescale, which can be of the order of the cavity lifetime ${\tau }_{c}$. This suggests the potential for photonic parallel information processing.

Furthermore, we proposed the application of a time-multiplexing encoding technique to wave dynamics and demonstrated enhancement in computational performance. This method could be used in situations when only a few detectors are available, owing to a physical constraint and, beyond the example of the microcavity lasers, the method will be applicable to any physical RC systems with spatial degree of freedom but only a few detectors.

Lastly, we discussed the sensing application options of the microcavity-based RC, in which case the microcavity is used as a sensing element as well as a reservoir. The combination of the optical sensing and RC could be used for the model-free identification of physical quantities. These results pave a way to utilize complex wave dynamical systems at microscale for fast photonic information processing and shed light on a potential trend toward model free sensing, using the concept of RC.

## Methods

### The Maxwell-Bloch model in microcavity lasers

We assume that the thicknesses of microcavities are much smaller than their in-plane dimensions, and that microcavities are treated as two-dimensional objects by applying effective refractive indices *n*_*in*_. To describe the light-matter interaction, we used the Maxwell-Bloch (MB) model where the gain medium inside the cavity is modelled as a two-level system. The Maxwell-Bloch model is a simple model to describe the laser dynamics, but it can qualitatively reproduce lasing phenomena in two-dimensional microcavity lasers^26,27^. The normalized Maxwell-Bloch model is given by

4 $$\frac{{\partial }^{2}E}{\partial {t}^{2}}=\frac{1}{\epsilon }{\nabla }_{xy}^{2}E-\sigma \frac{\partial E}{\partial t}-\Theta \frac{4\pi }{\epsilon }\frac{{\partial }^{2}}{\partial {t}^{2}}(\rho +c.c),$$

5 $$\frac{\partial \rho }{\partial t}=-\,({\gamma }_{\perp }+i{\Delta }_{a})\rho +{\gamma }_{\perp }WE,$$

6 $$\frac{\partial W}{\partial t}=-\,{\gamma }_{\parallel }(W-{W}_{\infty })-2iE{\gamma }_{\parallel }(\rho -{\rho }^{\ast }),$$

where space and time are made dimensionless by the scale transformations ${\omega }_{s}x/c\to x$ and ${\omega }_{s}t\to t$, respectively. ${\omega }_{s}$ is a reference frequency close to the transition frequency ${\omega }_{a}$ of the two-level gain medium. In Eqs. (4–6), *E*, $\rho$, *W* are the (dimensionless) electric field, polarization field, and population inversion component in a two-level gain system, respectively. All of the parameters are also made dimensionless. $\epsilon ={n}_{r}^{2}$ is the relative permittivity, where the refractive index *n*_*r*_ is *n*_*in*_ inside the cavity and waveguide, whereas ${n}_{r}={n}_{out}$ outside the cavity and waveguide. *σ* represents the background absorption inside the cavity. $\Theta (x,y)$ is a step function; $\Theta$ is 1 inside the cavity and zero outside the cavity, respectively. ${\Delta }_{a}={\omega }_{a}/{\omega }_{s}$ represents the normalized gain center. The two relaxation parameters, ${\gamma }_{\perp }$ and ${\gamma }_{\parallel }$, are the transverse and longitudinal relaxation rates, respectively. ${W}_{\infty }$ is the pumping power, representing an excitation power to the gain medium^26,27^. When ${W}_{\infty }=0$, the gain medium does not essentially affect the wave dynamics.

The Maxwell Eq. (4) was simulated using the finite-difference time-domain (FDTD) method, where a perfect matched layer (PML) was introduced near the boundary of the calculation space to absorb the emission light.

In the stadium cavity, shown in Fig. 1, the radius *R* of the half circle and major axis length $L=4R$ of the stadium were set $12.25/(\sqrt{2}{n}_{in})$ and $\mathrm{49/(}\sqrt{2}{n}_{in})$ in a unit of $c/{\omega }_{0}$, where ${\omega }_{0}$ is the input angular frequency, and *c* is the light velocity in vacuum. The actual *L* would be ~1.42 *μm* if ${n}_{in}=3.3$ and wavelength *λ* = 0.85 *μ*m. Although *L* is shorter than that of a standard microcavity, we restrict ourselves to the cases of short length owing to the lack of computational power. We emphasize that similar results can be essentially obtained in cases of longer length.

The values of the remaining parameters are ${n}_{in}=3.3$, $\sigma ={10}^{-3}$, ${\Delta }_{a}=1$, ${\gamma }_{\perp }=0.1$, ${\gamma }_{\parallel }={10}^{-4}$. *n*_*out*_ was set at 1 for the results shown in Figs. 1–6, whereas in Fig. 7, *n*_*out*_ is changed in a range of $1.3\le {n}_{out}\le 1.5$. We confirmed that these parameter values do not essentially affect the RC performance.

### Incident wave

The incident light *E*_*in*_ is phase-modulated with the input signal $u(n)$ (and mask signal when using the time-multiplexing method), and is injected into the cavity via the input waveguide shown in Fig. 1,

7 $${E}_{in}=A\psi \,\cos ({\omega }_{0}t+\phi (t)),$$

where *A* is the amplitude, $\psi$ is the lowest-order waveguide mode, $\phi (t)$ is the modulated phase, and ${\omega }_{0}$ is the center frequency of the input light. ${\omega }_{0}$ is tuned to the resonant frequency of the stadium cavity. The amplitude *A* is given such that the injection locking to the lasing mode with frequency ${\omega }_{0}$ is achieved when ${W}_{\infty }/{W}_{th} > 1$. All results presented in this paper are given under the injection locking condition.

The phase of the incident light is modulated as $\phi (t)={\psi }_{0}Mask(t)u(t)$, where ${\phi }_{0}$ is the modulation amplitude, *Mask*(*t*) and *u*(*t*) represents the mask signal and the input signal at time *t*, respectively. *u*(*t*) holds for a time interval ${\tau }_{s}\approx {\tau }_{c}=143/{\omega }_{0}$ or ${T}_{m}=M{\tau }_{s}$ when using the time-multiplexing method, where ${\tau }_{c}$ is the cavity lifetime. For a small cavity with $R=12.25/(\sqrt{2}{n}_{in})$ and input wavelength *λ* = 0.85 *μ*m, the cavity lifetime ${\tau }_{c}$ is less than 0.1 ps. Thus, sampling at an interval of ${\tau }_{s}\approx {\tau }_{c}$ is unrealistic. However, we remark that the problem can be moderated in a large cavity because ${\tau }_{c}$ can increase with increasing *R*^22^.

The mask signal *Mask*(*t*) is used only when the time-multiplexing method is applied, and it is given as a colored noise signal with a decay rate of $\mathrm{1/}{\tau }_{c}$, which is repeated with a time interval of *T*_*m*_. The use of such colored noise can efficiently excite the reservoir nodes used for the RC^35^.

### Estimation of parameter values toward actual experiments

In this study, we have restricted ourselves to a case concerning a small cavity of $L\approx 1.67\lambda$ because of the limitation of our computational power. We remark that the results in this paper have been presented in the normalized form, in which parameter values available for actual experiments can be estimated. For example, when *L* = 100 *μ*m, the average mode interval $\Delta {\omega }_{i}/(2\pi )$ will be in the order of gigahertz^28^. The photon lifetime ${\tau }_{c}$ in the stadium cavity (without a waveguide) is estimated as 28 ps and can be changed using amplification due to the gain medium. The sampling rate ${\tau }_{s}$ can be set as ${\tau }_{c}$ in an actual experiment. In addition, we also remark that, for a large cavity with *L* = 100 *μ*m, it is relatively easy to place the probes or single-mode waveguides coupled to detectors around the cavity.

### Consistency

Consistency is the similarity of the response outputs for a repeated drive signal and is considered one of the important properties of RC. The consistency can be measured in the correlation between the two response outputs, obtained from different initial conditions^31^. We measured the consistency of the wave dynamics in microcavity lasers driven by the input light *E*_*in*_ as follows:

8 $$C=\frac{1}{N}\mathop{\sum }\limits_{i=1}^{N}\,{C}_{i},$$

where

9 $${C}_{i}=\frac{\langle ({x}_{i}^{(1)}-{\bar{x}}_{i}^{(1)})\,({x}_{i}^{(2)}-{\bar{x}}_{i}^{(2)})\rangle }{{\sigma }_{i,1}{\sigma }_{i,2}}.$$

${x}_{i}^{(j)}(t)$ denotes the intensity signal detected by the probe *i*, which is obtained from an initial state *j*. 〈·〉 denotes the time average. ${\bar{x}}_{i}^{(j)}$ and ${\sigma }_{i,j}$ denote the time average and standard deviation of ${x}_{i}^{(j)}$, respectively. By the definition, *C* is in the range −$1\le C\le 1$ and takes the maximum $C=1$ when the two signals are identical, in other words ${x}_{i}^{(1)}(t)={x}_{i}^{(2)}(t)$.

## Supplementary information

Supplementary Information
