# Supplementary material for: Photonic reservoir computing based on nonlinear wave dynamics at microscale
Source: Sci Rep. 2019 Dec 13;9:19078. doi: 10.1038/s41598-019-55247-y (PMC6911076; doi:10.1038/s41598-019-55247-y)
Supplement: Supplementary file 1 — Supplementary Information [file 41598_2019_55247_MOESM1_ESM.pdf]

Supplementary information: Photonic reservoir computing based on nonlinear wave dynamics at microscale

Satoshi Sunada[1,2,*] **and Atsushi Uchida**[3]
[1] *Faculty of Mechanical Engineering, Institute of Science and Engineering, Kanazawa University Kakuma-machi Kanazawa, Ishikawa 920-1192, Japan*
[2] *Japan Science and Technology Agency (JST), PRESTO, 4-1-8 Honcho, Kawaguchi, Saitama 332-0012, Japan*
[3] *Department of Information and Computer Sciences, Saitama University, 255 Shimo-Okubo, Sakura-ku, Saitama City, Saitama, 338-8570, Japan.*

To further test the RC performance, we used two different types of tasks: nonlinear channel equalization tasks for classification and time-series prediction. In both tasks, we used 3000 training samples and 1000 test samples.

Nonlinear channel equalization.

A goal of this task is to reconstruct four digital signals $\{-3, -1, +1, +3\}$ transmitted through a noisy communication channel with nonlinear distortion. The nonlinear transformation of the communication channel is given by the following model equation [1, 2]:

$$\begin{aligned} q(n) = \quad & 0.08d(n+2) - 0.12d(n+1) + d(n) + 0.18d(n-1) \\ & -0.1d(n-2) + 0.091d(n-3) - 0.05d(n-4) \qquad (,1) \\ & +0.04d(n-5) + 0.03d(n-6) + 0.01d(n-7), \end{aligned}$$

$$u(n) = \quad q(n) + 0.036q(n)^2 - 0.011q(n)^3 + v(n), \qquad (,2)$$

where $d(n) \in \{-3, -1, +1, +3\}$ is the input signal with a random sequence, $q(n)$ is the linear channel output, $u(n)$ is the noisy nonlinear channel output, and $v(n)$ is the white Gaussian noise with a zero mean. In this task, $d(n)$ is recovered from $u(n)$. To perform the task, we used the time-multiplexing method explained in the main text. The output $\hat{y}(n)$ was obtained from the reservoir node responses to the input $u(n)$ by minimizing the error between $\hat{y}(n)$ and $d(n)$. An estimator $\hat{d}(n)$ was then obtained by replacing $\hat{y}(n)$ with a discrete value $\{-3, -1, +1, +3\}$ to which it is closest. The classification performance was evaluated with symbol error rate (SER).

Figure S1 shows SER as a function of signal-to-noise ratio (SNR) of the nonlinear channel signal. $N = 11$, $M = 5$ and $K = 0$ were used for the task. SER decreases with increase in SNR. SER also decreases by increasing the pumping power W_∞. At $W_\infty/W_{th} = 1.43$ and SNR = 30 dB, SER of 2×10^{-3} was obtained. This SER was better than that in case of the other compact photonic reservoir in [3], where the minimum SER of 2.2×10^{-2} was observed at the SNR of 31 dB. The SER will further improves for a larger-sized cavity and when N is increased.

We remark that the time-multiplexing method can be used to achieve good RC performance because the number of the effective nodes used to calculate the output $\hat{y}$ can increase to $NM(K+1)$. However, the maximum processing rate for classification is limited to $1/T_m = 1/(M\tau_s)$. In our case, the decrease in the processing rate, as M increases, can be moderated by using the spatial degree of freedom of a microcavity reservoir and increasing N, whereas the decrease in the rate is more significant in a reservoir without a spatial degree of freedom (e.g., a delay-based photonic reservoir [3]).

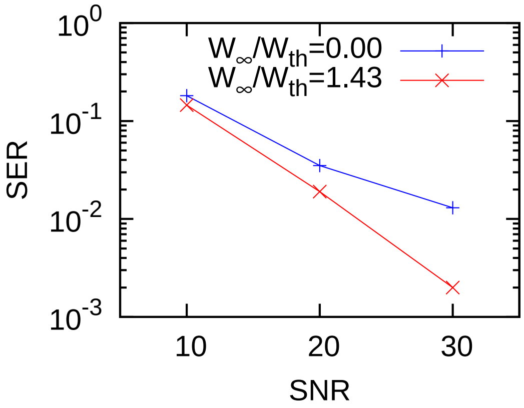

Figure S1: **Results of nonlinear channel equalization task.** The symbol error rate (SER) is plotted as a function of the signal-to-noise rate (SNR) of the nonlinear channel signal. The time-multiplexing method with $M = 5$ and $K = 0$ was used for this task. The SER decreases with increase in the SNR. The SER for $W_\infty/W_{th} = 1.43$ is better than the SER for $W_\infty/W_{th} = 0$ (without the gain).

Time-series prediction

We also performed the Santa Fe time-series prediction task [4], which is a single-point prediction task of chaotic data generated from a far-infrared laser. In this task, the input signal $u(n)$, which corresponds to the n-th sampling point of the chaotic waveform [Fig. S2a], $y(n)$, is used to predict the $n+1$-th sampling point, $y(n+1)$. The prediction error was evaluated using the normalized mean square error (NMSE), which is given by $1/T \sum_{n=1}^{T} [y(n) - \hat{y}(n)]^2/\sigma_y^2$, where σ_y^2 is the variance of the target signal $y(n)$.

Figures S2b shows the results of the prediction, where the time-multiplexing method with $M = 5$ and $K = 5$ were used. The NMSE at $W_\infty/W_{th} = 1.43$ was 0.117, whereas the NMSE at $W_\infty/W_{th} = 0$ was 0.332. The combination of the time-multiplexing method and an active gain can lead to good prediction performance. The NMSE of 0.117 is comparable to the NMSEs obtained for the other compact photonic RC (e.g., the NMSE of 0.109 in [3]).

References

[1] Jaeger, H. & Haas, H. Harnessing nonlinearity: predicting chaotic systems and saving energy in wireless communication. *Science* **304**(5667), 78-80 (2004).

[2] Paquot, Y. *et al.*Optoelectronic reservoir computing. *Sci. Rep.* **2**(1), 287 (2012).

[3] Takano, K. *et al.* Compact reservoir computing with a photonic integrated circuit. *Opt. Express* **26**(22), 29424-29439 (2018).

[4] Weigend, A. S. & Gershenfeld, N. A. Results of the time series prediction competition at the Santa Fe Institute, *IEEE International Conference on Neural Networks* **3**, 1786-1793 (1993).

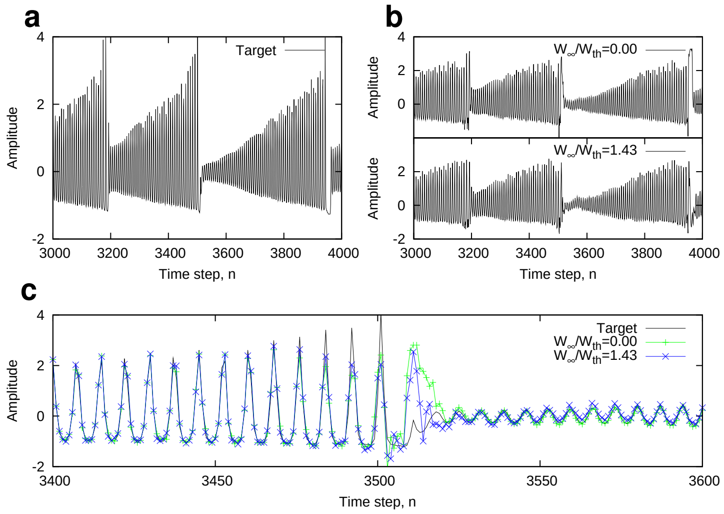

Figure S2: **Results of chaotic time-series prediction. a,** Target chaotic waveform signal. **b,** Reservoir outputs. The NMSEs are 0.332 and 0.117 for $W_\infty/W_{th} = 0$ and 1.43, respectively. **c,** Comparison between the target signal and reservoir outputs. Large prediction errors occur after the input signal level change around $n = 3500$.
